# TECHNOLOGICAL INFORMATION REGARDING PREBIOTICS AND PROBIOTICS NUTRITION VERSUS THE PATENT REGISTERS: WHAT IS NEW?

**DOI:** 10.1590/0102-6720201600040016

**Published:** 2016

**Authors:** José Maciel Caldas dos REIS, Maurício Fortuna PINHEIRO, André Takashi OTI, Denilson José Silva FEITOSA-JUNIOR, Mauro de Souza PANTOJA, Rui Sérgio Monteiro BARROS

**Affiliations:** 1 Master of Surgery and Experimental Research, State University of Pará - UEPA, Belém, PA, Brazil.

**Keywords:** INPI, Patents, Innovation, Foods.

## Abstract

**Introduction:**

Food is a key factor both in prevention and in promoting human health. Among the functional food are highlighted probiotics and prebiotics. Patent databases are the main source of technological information about innovation worldwide, providing extensive library for research sector.

**Objective:**

Perform mapping in the main patent databases about pre and probiotics, seeking relevant information regarding the use of biotechnology, nanotechnology and genetic engineering in the production of these foods.

**Method:**

Electronic consultation was conducted (online) in the main public databases of patents in Brazil (INPI), United States (USPTO) and the European Patent Bank (EPO). The research involved the period from January 2014 to July 2015, being used in the title fields and summary of patents, the following descriptors in INPI "prebiotic", "prebiotic" "probiotics", "probiotic" and the USPTO and EPO: "prebiotic", "prebiotics", "probiotic", "probiotics".

**Results:**

This search haven't found any deposit at the brazilian patents website (INPI) in this period; US Patent &Trademark Office had registered 60 titles in patents and the European Patent Office (EPO) showed 10 documents on the issue.

**Conclusion:**

Information technology offered by genetic engineering, biotechnology and nanotechnology deposited in the form of titles and abstracts of patents in relation to early nutritional intervention as functional foods, has increasingly required to decrease the risks and control the progression of health problems. But, the existing summaries, although attractive and promising in this sense, are still incipient to recommend them safely as a therapeutic tool. Therefore, they should be seen more as diet elements and healthy lifestyles.

## RESUMO

**Introdução:**

A alimentação é fator primordial tanto na prevenção quanto na promoção para a saúde humana. Dentre os alimentos funcionais destacam-se os probióticos e prebióticos. Os bancos de dados de patentes representam a maior fonte de informação tecnológica acerca de inovação em nível mundial, provendo vasta biblioteca para o setor de pesquisa.

**Objetivo:**

Realizar mapeamento nas principais bases de dados de patentes relacionada aos pré e probióticos buscando informações relevantes com relação ao uso da biotecnologia, nanotecnologia e engenharia genética na produção desses alimentos.

**Método:**

Foi realizada consulta eletrônica (online) nas principais bases de dados públicas de patentes do Brasil (INPI), Estados Unidos da América (USPTO) e o Banco de Patentes Europeu (EPO). A pesquisa envolveu o período de janeiro de 2014 a julho 2015, sendo utilizado nos campos de título e resumo das patentes, os seguintes descritores no INPI: "prebiótico", "prebióticos" "probiótico", "probióticos" e no USPTO e EPO: "prebiotic", "prebiotics", "probiotic", "probiotics"

**Resultados:**

Não foram observados, no INPI, depósitos de residentes (empresas ou universidades). Já no USPTO foram detectados 60 depósitos e no EPO 10 títulos de interesse à pesquisa.

**Conclusão:**

A tecnologia da informação ofertada pela engenharia genética, biotecnologia e nanotecnologia depositada na forma de títulos e resumos das patentes em relação à intervenção nutricional precoce como alimentos funcionais, tem cada vez mais pretendido diminuir os riscos e controlar a progressão de agravos à saúde. Mas, os resumos existentes, embora atraentes e promissores neste sentido, ainda são incipientes para recomendá-los de forma segura como ferramenta terapêutica. Portanto, devem ser encarados mais como integrantes de dieta e estilos de vida saudáveis.

## INTRODUCTION

The patent database is the largest source of technological information worldwide, providing extensive library for clinical, surgical or experimental research. Favors correlations in several areas and has a significant impact on the evolution of knowledge^6^ ^,^ ^7^.

Thus, the use of technology for the development of functional foods, biologically active, becomes increasingly challenging, as it seeks to meet the consumer demand for products concomitantly healthy, attractive and nutritious^4^ ^,^ ^10^.

So, food is a key factor both in prevention and in promoting human health. Among the functional food are highlighted probiotics and prebiotics, or foods that have beneficial health effects and may also be boosted with their association, resulting in so-called symbiotic effect^4^ ^,^ ^9^. 

The World Health Organization defined probiotics as "Live microorganisms which when administered in adequate amounts confer health benefits for the host". They are inactivated substances that act as energy supplement for beneficial bacteria. Prebiotics "are indigestible ingredients that benefit the host by selective stimulation of the growth and/or activity of one or a limited number of bacteria in the colon". On the other hand, symbiotics are combinations of prebiotics and probiotics, so that a symbiotic product exerts both a prebiotic and probiotic effects^4^ ^,14^.

Therefore, there are several factors that have stimulated the development of so-called functional foods over the past few years. Likewise, there was increased use of biotechnology, nanotechnology and genetic engineering in the production of these foods. It was mainly driven by an increase in life expectancy in developed countries (whose populations require hospital care for a longer period of time), the high cost of health services, the need that public research institutions have to disclose the results of their investigations and advances in food technology and ingredients^9^ ^,^ ^10^.

 The patent databases, although available online for free, are unexplored as technological information providers documents, both for educational and research institutions such as universities^1^ ^,^ ^2^ ^,^ ^5^ ^,^ ^13^. 

Thus, this article aims to analyze the use of technological information based on patent records (titles and abstracts) available both within the country and internationally, checking the deposited advances and direction in relation to pre and probiotics. 

## METHOD

The universe chosen to be part of this study was bibliographic quantitative and qualitative, retrospective and cross from electronic consultation (online) in the main public databases of patents in Brazil (Instituto Nacional da propriedade Industrial - INPI)^7^, United States (The United States Patent and Trademark Office - USPTO)^13^ and the European Patent Database (European Patent Office - EPO)^2^, covering patents filed and published in more than 90 countries, including Brazil, available for free (www.espacenet.com). 

The research involved a maximum of 19 months (including the dates of deposits from January 2014 to July 2015) and was based methodologically in the fields of spreadsheets regarding the titles and abstracts of patents across the following descriptors: on INPI "prebiótico", "prebióticos" "probiótico", "probióticos"; on USPTO and EPO "prebiotic", "prebiotics", "probiotic", "probiotics". Data collection from the titles and abstracts of patent documents was focused on documents that made reference to the protected technology (products, information and processes). To interpret the information of interest, each document was analyzed individually by identifying the number of patents found in each base, and their collected relevant information describing inventions, categorizing the type of depositor, the country and the sector to which apply the invention and relevance for functional foods.

Inclusion criteria were the titles and abstracts of each patent process about innovation with new technologies in probiotics and prebiotics, ie. products and processes that alluded to the above terms genetic engineering, nanotechnology and biotechnology and new substances with a focus on therapy in humans, such as pre supplementation or postoperative and treatment of neoplastic diseases or other conditions or health problems. The titles or files relating to experimental treatments in animals, veterinary supplementation therapies, food products not related to medical therapy or other species of pre- or probiotics unreferenced for clinical use were excluded.

To interpret the information of interest, each document provided the relevant describing inventions, their contribution to the current clinical treatment and the potential for improvements in the treatment or control of diseases in humans. In the virtual consultation were used available tools in each homepage (INPI, USPTO e EPO)^1^ ^,^ ^2^ ^,^ ^5^ ^,^ ^13^. 

### Statistical analysis

The selection was made through filters applied to worksheets in Microsoft Excel, eliminating duplicate documents between the centers, as different countries are subject to the laws of different industrial properties^1^ ^,14^. The results were analyzed in simple descriptive statistic and highlighted about their contributions to the current medical therapy.

## RESULTS

Does not exist on the INPI any resident deposits (companies or universities) related to the selected scopes. In the USPTO were found 480 records, and after filtering parameters of interest of the research, it was found 60 deposits. As for the European Patent Office, EPO were found 426 deposits, after the use of filters in Microsoft Excel spreadsheets, remained 10 titles of interest to the research.

In Brazil, during the period established for the study of this article, we found no title or process related to probiotics and prebiotics terms, or their terms in the singular, even deepening the search tools on the filters, including the titles and their respective summaries. Therefore, especially for developing countries, such as Brazil, the information contained in a patent document should lead to the identification of alternative and emerging technologies and market trends, assisting in making investment decisions and opportunities. Nevertheless, even not the case of the proposed methodology, it was found that the most recent record was up to European product registered in Brazil and dated March 18, 2013 (EPO 12161083.6)^5^.

Regarding the US Patent & Trademark Office (USPTO) a larger number was identified, totaling 4,200 records; however, the summary search tool is not accurate in determining the terms of medical therapies, human genetics, surgical supplementation and bio or nanotechnology. Therefore, further research of the terms and summaries of each title was necessary to select those intended in this paper^13^. Was reached, finally, an approximate number of 480 patent records related to medical therapy in the USPTO. There is the proposal of micro, nano and submicron structures with Amaranth protein combined with at least one other biopolymers for packaging and carrying of medicines^13^. It was found around 60 records therapies relating the pre and probiotics to treat diarrhea and inflammatory bowel disease for symptom relief and restoration of intestinal microflora^13^. Thus, the products called pre and probiotics, have grown in recent years. American Dietetic Association data revealed that more than 80% of Americans consume or would like to consume functional food and/or beverages^6^. In Latin America the market for probiotic yogurts grew 32% only in the 2005-2007 period, demonstrating growth since that time. In Brazil, there are no accurate estimates of the market for these products; however, it is known that US consumers annually invests $90 in functional food and drinks^9^.

We highlight the inventions of probiotics for nutrition in pre and postoperative operations of the colon, where it is observed frequent association of inventors and large industry companies (Schffrin USPTO 20130344044, Mark Pimentel USPTO 9,066,962). Also in relation to nutrition was deposited by private enterprise association (Nestec S.A) e Darimont (inventor) the use of probiotics to treat obesity with *Lactobacillus rhamnosus* CGMCC 1.3724 and/or *Lactobacillus rhamnosus* NCC4007 for maintenance and weight loss, treating obesity^13^.

Relative to the US patent office (USPTO) was observed record by Kibow Biothech Inc. using prebiotics with extract of *Lentinula edodes mycelia* and polyphenol *Litchi chinensis fruit* to improve the effectiveness of radiation and chemotherapy in the treatment and prevention of colorectal cancer (USPTO 9,072,768). It was also highlighted the deposit found which lists the dextrans as probiotics and replicated through genetic engineering to changing dextransucrases maintaining its enzymatic activity and specificity of synthesis of .alpha -1.6 bonds^13^.

Concerning the Espacenet (European Patent Office, EPO) deposits found on prebiotics and probiotics contemplated an initial number of 426 patent titles with reference to the proposed period of 19 months (2014 and 2015)^2^. The data search system has more didactic accessibility tools in relation to the period and summaries are most notably of the terms set. Initially gained prominence pharmaceutical proposal of Dorozhko Oleg Valentinovich (EPO- SI2415475(T1) associating a prebiotic that stimulates the growth of *Lactobacilli* (antagonist of *H.pylori*) in the upper gastrointestinal tract, which in combination with a proton pump inhibitor alters gastric pH and promotes eradication of *H.pylori*. Thus, it provides definitive and lasting treatment for gastric and duodenal ulcer reducing the incidence of recurrences^2^.

It was observed in 10 titles the probiotic relation to the treatment of irritable bowel syndrome and inflammatory bowel diseases; in this context, we emphasize the deposit authored by Fiore Esteban Alejandro (MX2013002737-A) associating trimebutine, propinox, lansoprazole, cinitapride, ranitidine and famotidine to the probiotic compound of *Lactobacillus acidophilus* for oral administration in order to restore the intestinal function^2^. The inventor Wang Gary Ping obtained patent rights for specific treatment of infection by *Clostridium difficile* using bacteria as probiotics elements^2^. 

Genetic engineering in combination with probiotic micro-organisms is designed for the treatment of gastrointestinal inflammatory diseases. It has been proposed use of transgenic probiotic micro-organism genetically engineered to express and secrete Cystatina with the corresponding amino acid sequence SEQ ID NO 1 to 3 for the treatment of intestinal disorders^2^. Other inventions related to pro and prebiotics are highlighted the treatment of diarrhea, laxatives, immunity enhancement through the intestinal microbiota and control of intestinal disorders^2^.

In the field of therapeutic dermatology and cosmiatry were designed probiotics with *Lactobacillus plantarum* HY7714 for treating wrinkles and moisturizing (KR20140128674-A, WO2015106175, US2015202136 e EP2704704); in allergy and immunology was found record of probiotics and prebiotics products that reduce the crises, the eosinophilia and increase resistance and immunity^2^ ^,^ ^12^.

In the field of biotechnology and nutrition attracted attention the invention project about gelatinous mixture of prebiotic and probiotic compound for the treatment of chronic renal failure; these compounds, according to the inventors, reduce the concentration of uremic toxins and improve kidney function by reducing water retention. The said compound uses live and active bacteria in vegetable prebiotic fibers^2^. Finally, it is worth emphasizing the project US2014147426 related to oral health, where bacteria of the oral cavity of individuals without caries were isolated and is seeking to identify their anti-caries protective factors^2^. Consequently, compounds have been developed in several pharmaceutical forms (toothpaste, oral solutions, chewing gum and others) with the respective peptides anti-caries artificially developed. Thus, biotechnology has an increasingly vital role in food production and, in the field, is increasingly growing the use of animals and plants for the food industry. Thus, it is clear that biotechnology is not directed only to food production, but it is also important tool to meet the consumer demand for a safe and healthy product^2^ ^,^ ^3^ ^,^ ^8^ ^,^ ^11^ ^,^ ^13^.

Patents under intellectual property are important for two reasons. First, because they constitute the most tangible form of intellectual property and enjoy strong legal protection. Second, because the patent databases are powerful sources of technological information to be used in competitive intelligence. Therefore ever more must be encouraged the undergraduate and graduate environment to exploit this source of knowledge^1^ ^,^ ^5^. In Brazil, there is still a need to expand investment and protection of industrial property to generate new products, because the number of patents is still incipient. There is need for greater government incentive to create partnerships between business and research centers.

## CONCLUSION

Information technology offered by genetic engineering, biotechnology and nanotechnology deposited in the form of titles and abstracts of patents in relation to early nutritional intervention as functional foods, has increasingly required to decrease the risks and control the progression of health problems. But, the existing summaries, although attractive and promising in this sense, are still incipient to recommend them safely as a therapeutic tool. Therefore, they should be seen more as diet elements and healthy lifestyles.

## References

[1] Brasil (1996). Lei nº 9.279.

[2] EPO (2010). Base de dados contendo documentos de patente dos países europeus.

[3] FAO/OMS (2006). Probiotics in Food. Health and Nutritional Properties and Guidelines for Evaluation.

[4] Ferreira CLLF, Ferreira CLLF (2012). Grupo de bactérias lácticas e aplicação tecnológica de bactérias probióticas.

[5] Instituto Nacional de Patentes Industriais [online] (1970). Instituto Nacional de Patentes Industriais.

[6] Lemos R (2005). Propriedade Intelectual.

[7] Flesch AG, Poziomyck AK, Damin DC (2002). Oliveira MN, Sivieri K, Alegro JH, Saad SM Aspectos tecnológicos de alimentos funcionais contendo probióticos. São Paulo. Rev Bras Ciênc Farm.

[8] Saad SMI, Komatsu TR, Granato D, Branco GF, Buriti FCA, Saad SMI, Cruz AG, Faria JAF (2011). Probióticos e prebióticos em alimentos: aspectos tecnológicos, legislação e segurança no uso. Probióticos e prebióticos em alimentos: fundamentos e aplicações tecnológicas.

[9] Santos FL, Ferreira MA, Pires EA, Oliveira FS, Silva CFG, Vieira RB (2014). Análise das patentes de tecnologias relacionadas aos probióticos, prebióticos e simbióticos no Brasil Braz. J. Food Technol.

[10] Simon O, Jadamus A, Vahjen E (2001). Probiotic feed additives - effectiveness and expected modes of action, Journal of Animal Feed. Science.

[11] Souza FS, Cocco RR, Sarni ROS, Mallozi MC, Solé D (2010). Prebióticos, probióticos e simbióticos na prevenção e tratamento das doenças alérgicas. Rev Paul Pediatr.

[12] (2012). The United States Patent and Trademark Office.

[13] Varavallo MA, Thome JN, Teshima E (2008). Aplicação de bactérias probióticas para profilaxia e tratamento de doenças gastrointestinais. Semina: Ciências Biológicas e da Saúde.

